# A genetic toolkit for the analysis of metabolic changes in *Drosophila* provides new insights into metabolic responses to stress and malignant transformation

**DOI:** 10.1038/s41598-019-56446-3

**Published:** 2019-12-27

**Authors:** L. Gándara, L. Durrieu, C. Behrensen, P. Wappner

**Affiliations:** 1 Instituto Leloir, Ciudad de Buenos Aires, Argentina; 2 Departamento de Fisiología, Biología Molecular, y Celular, Facultad de Ciencias Exactas y Naturales, Universidad de Buenos Aires, Ciudad de Buenos Aires, Argentina; 3 Consejo Nacional de Investigaciones Científicas y Técnicas (CONICET), Ciudad de Buenos Aires, Argentina

**Keywords:** Cell biology, Biological techniques

## Abstract

Regulation of the energetic metabolism occurs fundamentally at the cellular level, so analytical strategies must aim to attain single cell resolution to fully embrace its inherent complexity. We have developed methods to utilize a toolset of metabolic FRET sensors for assessing lactate, pyruvate and 2-oxoglutarate levels of *Drosophila* tissues *in vivo* by imaging techniques. We show here how the energetic metabolism is altered by hypoxia: While some larval tissues respond to low oxygen levels by executing a metabolic switch towards lactic fermentation, the fat body and salivary glands do not alter their energetic metabolism. Analysis of tumor metabolism revealed that depending on the genetic background, some tumors undergo a lactogenic switch typical of the Warburg effect, while other tumors do not. This toolset allows for developmental and physiologic studies in genetically manipulated *Drosophila* individuals *in vivo*.

## Introduction

**Figure 1:**
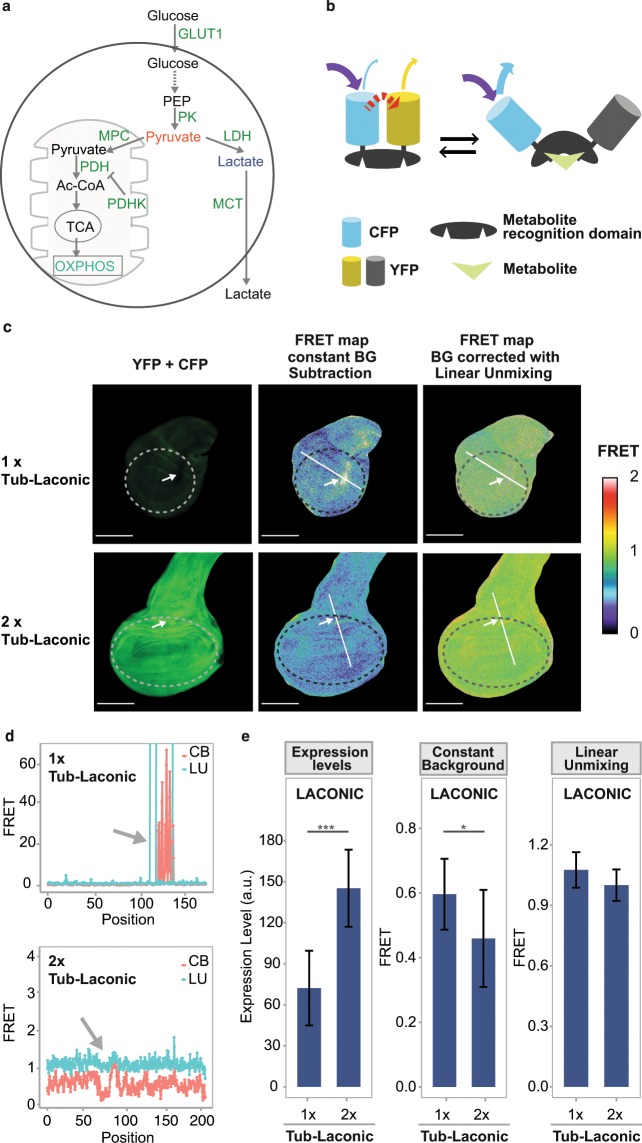
Sensors for studying cellular bioenergetics. (**a**) Diagram of glucose catabolism. Glucose is broken down at glycolysis and then can be fully oxidized to CO_2_ at the mitochondria (left), or alternatively, partially oxidized to lactate (right); pyruvate (red) stands as the branching point between the two alternative pathways. (**b**) Schematic representation of the FRET sensors^12–14^. The donor and acceptor fluorophores, CFP and YFP, are represented in blue and yellow, respectively. Binding of the corresponding metabolite (green) to its binding domain elicits a conformational change that separates CFP from YFP, and FRET ceases. (**c**,**d**) FRET maps of the Laconic signal of wing imaginal discs in which different expression levels of the sensor were attained by introducing either 1 or 2 copies of the tub-Laconic construct. Note that the apparent FRET signal obtained after subtracting a constant background value is dependent on the expression levels of the sensor, while this dependency is largely suppressed after applying the linear unmixing algorithm (compare 1x vs 2x Tub-Laconic and the region pointed by arrows to the rest of the tissue). A high FRET signal in the color code shown on the right must be associated to low lactate levels. Scale bar: 100 μm. Dotted lines show the region in which the FRET signal was measured. (**e**) Quantification of Laconic expression and of the FRET signal obtained after constant background subtraction (CB) or applying the linear unmixing algorithm (LU) in wing discs with 1 or 2 copies of Tub-Laconic. Data represent the media +/− SD; p = 1.4292E-05 for expression levels; p = 0.0358 for FRET signal (LU); Student’s T-test; n ≥ 20 per group.

Carbohydrate catabolism is at the core of cellular bioenergetics^1^. Pyruvate originated from glycolysis can be either reduced to lactic acid, or enter the mitochondria, where it is further oxidized to CO_2_ through the Krebs cycle reactions, providing reduced co-factors such as NADH or FADH_2_ that feed the electron transport chain, which generates the driving force for ATP synthesis *via* oxidative phosphorylation (OXPHOS)^1^. Cells need to balance lactic fermentation and OXPHOS to cope with energetic and anabolic requirements upon changes in the environment. For example, mitochondrial OXPHOS becomes largely suppressed in hypoxia, as has been described in many models^2–5^. To cope with this altered cellular physiology, many cells are capable of decoupling carbohydrate catabolism from mitochondrial OXPHOS by reducing pyruvate to lactate (Fig. 1a). This metabolic status can be achieved through the regulation of a few enzymes or transporters that, acting together, control the metabolic flux. The main enzymes involved in this rewiring are *lactate dehydrogenase* (LDH), which converts pyruvate into lactate^1^, and *pyruvate dehydrogenase kinase* (PDHK), which prevents pyruvate conversion into acetyl-CoA through the inhibition of the Pyruvate Dehydrogenase complex^1^. Both enzymes, LDH and PDHK, are transcriptionally upregulated in hypoxia^6,7^. Likewise, other environmental challenges, such as nutrient deprivation^1^ or osmotic shock^8^, can also alter the metabolic profile of the cell.

Several analytical methodologies are currently utilized to study metabolism in cell culture or animal tissues, provided sufficient amounts of material are available for preparing homogenates to perform biochemical analyses. These techniques encompass colorimetric assays to monitor enzymatic activity^9^, chromatography (either GC, HPLC or UPLC) followed by mass spectrometry, or nuclear magnetic resonance to measure concentrations of different metabolites^10^, as well as devices to study mitochondrial activity by assessing oxygen consumption rates^11^. However, none of these widely used methodologies can monitor metabolic parameters in an intact organism with spatial resolution. The recent development of genetically-encoded fluorescent metabolic sensors opens this possibility, but imaging and data processing methods need to be improved to obtain reliable results in whole organisms or tissues.

Three FRET sensors that report levels of lactate (Laconic)^12^, pyruvate (Pyronic)^13^ or the Krebs cycle metabolite 2-oxoglutarate (2-OG) (OGsor)^14^ have been developed in simple model systems such as bacteria or cell culture. In all three sensors, binding of the corresponding metabolite elicits a conformational change that separates the donor from the acceptor fluorophore, preventing resonant energy transfer (Fig. 1b). Two of these sensors, Laconic and Pyronic, were then adapted to mice^15^ and flies^16^, although they have been utilized to monitor metabolic changes within single cells rather than to compare the metabolic status of a cell with that of the rest of the tissue^12,13^. We have independently generated transgenic fly lines for these three sensors, however when we tried to assess metabolite levels in individual cells in the context of intact organs, we faced artefactual results that precluded comparison between cells of endogenous metabolite levels. We present here an image processing method to obtain reliable FRET signals from the metabolic sensors Laconic, Pyronic and OGsor, opening the possibility to assess metabolite levels in developmental or physiologic studies in intact tissues or whole *Drosophila* organs. We employed these sensors to compare the metabolic responses to hypoxia in different tissues, revealing that not all tissues undergo a lactogenic switch in the same manner. We also analyzed the occurrence of the Warburg effect in different experimental tumors induced in *Drosophila* larvae, and found that this metabolic rewiring depends highly on their genetic background. We show that the tools and methods presented here can provide qualitatively distinct information to the biochemical approaches widely used in the field.

## Results and Discussion

### Optimized analysis of FRET sensor signal in full organs

#### Image processing algorithm to deal with autofluorescence

We generated fly lines in which the sensors Laconic, Pyronic or OGsor are expressed under control of UAS sequences, as well as a line that expresses Laconic ubiquitously under control of a Tubulin promoter (Methods). Initial attempts to employ these tools in whole *Drosophila* organs were unsuccessful due to diverse imaging artefacts: Most notably, the Laconic FRET signal seemed to correlate with expression levels of the sensor (Fig. 1c–e). Induction of increasing levels of Laconic expression in 3^rd^ instar larval wing imaginal discs brought about an apparent FRET signal that tightly correlated with expression levels of the sensor (Fig. 1c–e and Supplementary Fig. 1a–e). In reporters such as these sensors, in which the donor/acceptor pair is part of the same protein (intramolecular FRET), the FRET signal should not depend on the sensor concentration in the sample, but only on their bound-to-unbound average ratio^17^. This artefactual dependency on sensor expression levels prevents the use of the sensors in studies where different cells or tissues need to be compared, as expression levels of transgenes (the FRET sensors in this case) are never fully homogeneous. As a possible cause of this artefactual behavior, we noticed that *Drosophila* larval tissues display high degree of pixel-to-pixel autofluorescence heterogeneity (Supplementary Fig. 1f). Thus, background correction by subtraction of an average autofluorescence constant value, while effective in cell culture, is inappropriate *in vivo* (Fig. 1c,d, arrows).

To cope with autofluorescence heterogeneity, we adapted a *linear unmixing algorithm*^18^, which estimates the autofluorescence contribution to the signal with single-pixel resolution (*Supplementary Information*). Briefly, the problem stems from the impossibility to estimate this heterogenic autofluorescence contribution to each pixel from an image of either the YFP or CFP channel. The linear unmixing algorithm allows dissecting these fluorescence sources, using a dedicated third image acquired in an emission window where only autofluorescence can be detected (Supplementary Fig. 1g). After applying the linear-unmixing algorithm, the Laconic FRET signal does not depend any longer on sensor expression levels (Fig. 1c–e and Supplementary Fig. 1a–e).

**Figure 2:**
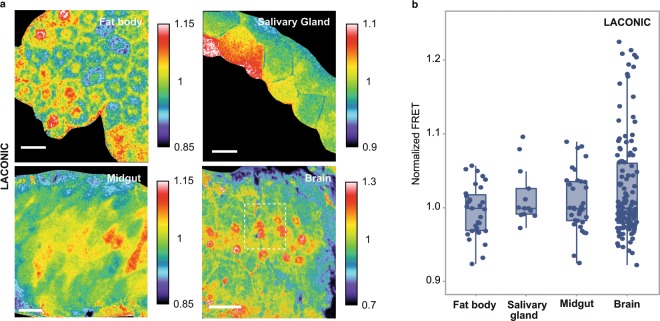
Laconic reveals single-cell variations of stationary lactate levels. (**a**) Laconic FRET maps of a fat body, salivary gland, larval midgut (alkaline region, next to the midgut/hindgut junction) and larval brain (ventral nerve cord). In all four organs, cells with various different endogenous levels of lactate can be distinguished. In the brain sample, the specific area marked with a dotted line is analyzed in more detail in Supplementary Fig. 2. Scale bar: 50 μm. (**b**) The points represent the average FRET signal of each cell shown at the images of panel (a). Data distribution is represented by the box and whiskers graph.

In this manner, calculation of the FRET signal for each pixel defines a FRET map of a given 3^rd^ instar larval organ (Fig. 2a). Analysis of different *Drosophila* larval tissues with this method revealed subtle variations of lactate concentrations amongst individual cells (Fig. 2a,b). The comparison between the emission spectra of cells with different Laconic signals in the larval brain reveals that changes of donor and acceptor emission intensities are inversely correlated (Supplementary Fig. 2). This confirms that the cell-to-cell differences in FRET signals reported here are not artefactual, and probably reflect variations of intracellular lactate levels.

Thus, by utilizing these sensors with a linear unmixing algorithm, the metabolic status of individual cells in the context of whole *Drosophila* organs can be assessed.

#### Validation and characterization of the FRET signal

As a next step, we confirmed that energy transfer from the donor to the acceptor does indeed occur in our experimental setting. An assumption in FRET experiments is that the donor excitation wavelength does not induce direct excitation of the acceptor, and thus that all the fluorescence emission of the acceptor originates from energy transferred from the donor (Fig. 1b). We therefore utilized a transgenic line that expresses only YFP (the acceptor) to rule out that the excitation wavelength of the donor (458 nm) can induce direct excitation of the acceptor in this experimental setting. As expected, this was not the case, implying that the YFP signal detected in flies bearing the sensor does indeed arise from FRET (Sup Fig. 1h).

As an additional control that FRET happens in transgenic flies carrying the sensors, we performed a set of *acceptor photobleaching* assays. With each of the sensors, we photoinactivated the acceptor fluorophore (YFP) by irradiating at high fluence a defined area of a larval wing imaginal disc with a wavelength at which the donor (CFP) is not stimulated (488 nm). If FRET does indeed occur, an increased emission of the donor is expected from the photobleached area upon irradiation at its excitation wavelength (458 nm). Comparison of the Laconic emission spectra of the irradiated region before and after YFP bleaching revealed an increase in CFP fluorescence (Sup Fig. 1i,j), confirming that resonant energy transference effectively occurs between donor and acceptor. For Pyronic, lower fluence doses were employed to prevent undesired CFP photoinactivation, and in these conditions an increment of CFP fluorescence after photobleaching could be measured, which validates the Pyronic FRET signal (Sup Fig. 1k). The YFP photobleaching assay on OGsor-expressing tissues only led to increased CFP emission in non-fixed material (Sup Fig. 1l), so the experiments involving OGsor were carried out in live organs.

**Figure 3:**
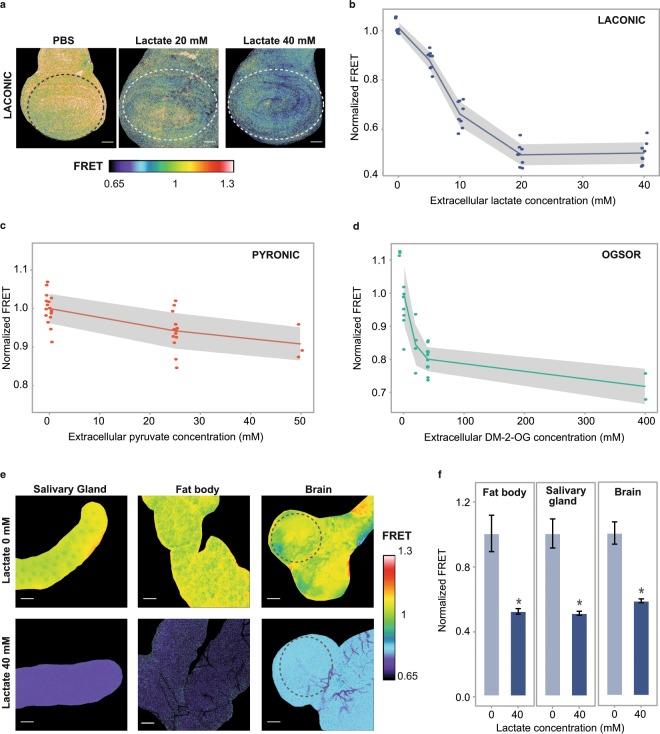
Response of the sensors to exogenously supplied metabolites. (**a**) Laconic FRET maps of wing imaginal discs incubated or not in 20 mM or 40 mM lactate for 15 min. Scale bar: 50 μm. Dotted lines mark the region in which the FRET signal was measured. (**b**) Quantification of the Laconic signal of the experiment of panel a. Each point represents the average value of a single imaginal disc. The grey area is limited by the SD of each data set. (**c**) Pyronic FRET signal from wing imaginal discs incubated or not with exogenous pyruvate for 15 min. Each point represents the average value of a single imaginal disc. The grey area is limited by the SD of each data set. (**d**) OGsor FRET signal from wing imaginal discs incubated or not with exogenous DM-2-OG for 15 min. Each point represents the average value of a single imaginal disc. The grey area is limited by the SD of each data set. (**e**) FRET maps of the indicated 3^rd^ instar larval organs with or without addition of exogenous 40 mM lactate. Dotted lines mark the region in which the FRET signal was measured. Scale bar: 50 μm. (**f**) Quantification of the Laconic signal of the experiment shown in panel (e). Data represent the media +/− SD; p = 2.27E-08 for fat body, p = 1.0442E-09 for salivary gland, p = 3.596E-11 for brain; Student’s T-test; n ≥ 20 per group.

Next, to define the concentration range at which each of the sensors responds to its corresponding metabolite, we performed *ex-vivo* experiments. Wing discs dissected from Laconic or Pyronic-expressing larvae were incubated in PBS buffer with increasing concentrations of lactate or pyruvate encompassing the expected physiological range, which rarely exceeds 10 mM^19,20^. Both sensors reported a reduction of the FRET signal proportional to the concentration of the corresponding metabolite (Fig. 3a–c). Since 2-OG does not diffuse across the plasma membrane, to test OGsor responses we utilized the membrane permeable analog dimethyl-2-oxoglutarate (DM-2-OG). After reaching the cytosol, DM-2-OG is demethylated and converted into 2-OG, increasing its intracellular levels^21^ and altering the OGsor FRET signal (Fig. 3d). Other larval organs such as brains, fat bodies and salivary glands expressing Laconic showed a similar behavior upon incubation with lactate (Fig. 3e,f). The specificity of this response was assessed by incubating sensor-expressing wing discs in solutions of a metabolite to which a given sensor is not supposed to respond. As expected, all three sensors responded in a metabolite-specific manner (Supplementary Fig. 1m–o).

### Environment-induced metabolic changes

**Figure 4:**
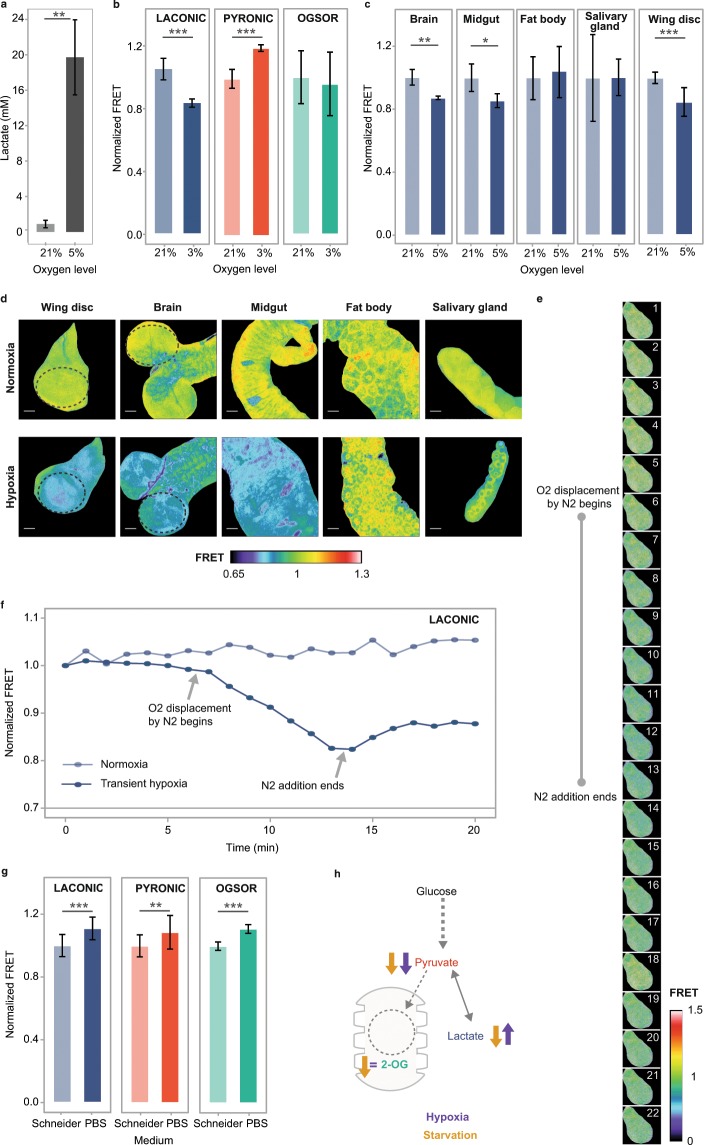
Metabolic rewiring upon O_2_ or nutrient deprivation. (**a**) Colorimetric determination of lactate in homogenates of whole larvae subjected or not to hypoxia for 16 h. Data represent the media +/− SD; p = 0.0078; Student’s T-test. n = 4 per group. (**b**) Laconic, Pyronic and OGsor FRET signal from wing imaginal discs from larvae exposed or not to hypoxia for 16 h before dissection and observation. Data represent the media +/− SD; p = 8.1723E-06 for Laconic and p = 6.878E-05 for Pyronic; Student’s T-test. n ≥ 20 per group. (**c,d**) Laconic FRET maps and quantification of wing discs, brains, midguts, fat bodies and salivary glands of 3^rd^ instar larvae exposed or not to hypoxia. Note that the imaginal discs, brain and midgut from larvae exposed to hypoxia increase their lactate levels, while salivary glands and the fat body do not. Dotted lines mark the region in which the FRET signal was measured. Scale bar: 50 μm. Data represent the media +/− SD; p = 0.0009 for wing discs; p = 0.0011 for brain and p = 0.0494 for midgut; Student’s T-test; n ≥ 20 per group. (**e,f**) Time course of Laconic signal variation in a representative wing imaginal disc exposed or not to transient hypoxia. (**g**) Laconic, Pyronic and OGsor FRET signal of 3^rd^ instar larvae wing imaginal discs incubated for 20 min in either Schneider medium or PBS prior to confocal analysis; data represent the media +/− SD; p = 2.5507E-06 for Laconic, p = 0.0037 for Pyronic and p = 4.1068E-05 for OGsor; Student’s T-test; n ≥ 20 per group. (**h**) Scheme of carbohydrate catabolism; variations of the metabolites monitored in this study upon hypoxia or starvation are indicated.

Stress conditions are known to alter the cellular energetic metabolism^1,22,23^, so we began by assessing the FRET signal of the three sensors in hypoxia. By using a colorimetric assay in whole-larvae homogenates, we confirmed that oxygen deprivation induces lactate accumulation (Fig. 4a), although with this method it is not possible to assess this metabolic switch with spatial resolution. Thus, we explored if by using the FRET sensors we can analyze metabolic responses to hypoxia in different larval organs. Wing discs of larvae exposed to hypoxic conditions for 16 h displayed increased lactate and decreased pyruvate levels, while we did not observe changes in the concentration of 2-OG (Fig. 4b). These observations indicate that a lactogenic switch occurs in wing discs of hypoxic larvae. Interestingly, while the larval brain and midgut also executed a lactogenic switch, salivary glands and the fat body did not (Fig. 4c,d).

Metabolic changes must occur rapidly, so that cells can maintain homeostasis in a quick shifting environment. To investigate the dynamics of this metabolic adaptation to hypoxia, Laconic-expressing wing imaginal discs were exposed to hypoxia *ex-vivo*, while recording their FRET signal under the confocal microscope. We observed that very rapidly after the onset of hypoxia exposure, a reduction of the FRET signal can be detected, while later on, as the hypoxic condition persists, the Laconic FRET signal continues to decrease. (Fig. 4e,f and Supplementary Video 1).

Nutrient deprivation is another environmental condition that can alter cellular bioenergetics. Starved *Drosophila* larvae experience a sharp reduction of catabolism of carbohydrates, proteins and lipids^22^. This global metabolic constriction is thought to play a role in starvation resistance, albeit strong evidence supporting this hypothesis is still elusive^24,25^. The way in which starvation-induced catabolic inhibition affects the stationary levels of the metabolites analyzed here has not been explored at the cellular level, although lactate concentrations in the whole larva have been shown to diminish upon protein starvation^26^. Larvae expressing each of the three sensors were subjected to 6 h starvation, a condition strong enough to induce autophagy^27^. No alterations of the FRET signal from any of the sensors could be detected (Supplementary Fig. 3a). These results suggest that larval starvation does not impinge on intracellular levels of lactate, pyruvate or 2-OG, probably due to physiological compensation mechanisms capable of maintaining cellular homeostasis. The Laconic signal also remained unaltered upon a longer (18 h) starvation period in all of the analyzed organs (Supplementary Fig. 3b). Noteworthy, these tools cannot report on every kind of metabolic rewiring: only those cases that lead to altered steady-state levels of the monitored metabolites (lactate, pyruvate or 2-OG) are visible to the sensors, while compensated flow changes might still occur.

If systemic compensation mechanisms do indeed account for intracellular stability of lactate, pyruvate and 2-OG steady-state levels, variations of these metabolites might be observed in tissues subjected to starvation *ex vivo*. We incubated wing imaginal discs dissected from larvae in Schneider (rich) medium or in PBS (extreme starvation) for 15 minutes. Starved imaginal discs displayed decreased levels of lactate, pyruvate and 2-OG, as reported by each of the sensors (Fig. 4g). Thus, an overall nutrient restriction results in reduction of all three metabolites, which probably reflects the decreased metabolic flux characteristic of the starvation response.

In summary, whilst hypoxia induces a lactogenic switch in some tissues that involves increased lactate and reduced pyruvate levels, extreme starvation leads to decreased concentrations of the three metabolites, lactate, pyruvate and 2-OG, reflecting an overall reduced metabolic flux (Fig. 4h).

### Genetic manipulations of the bioenergetic metabolism

**Figure 5:**
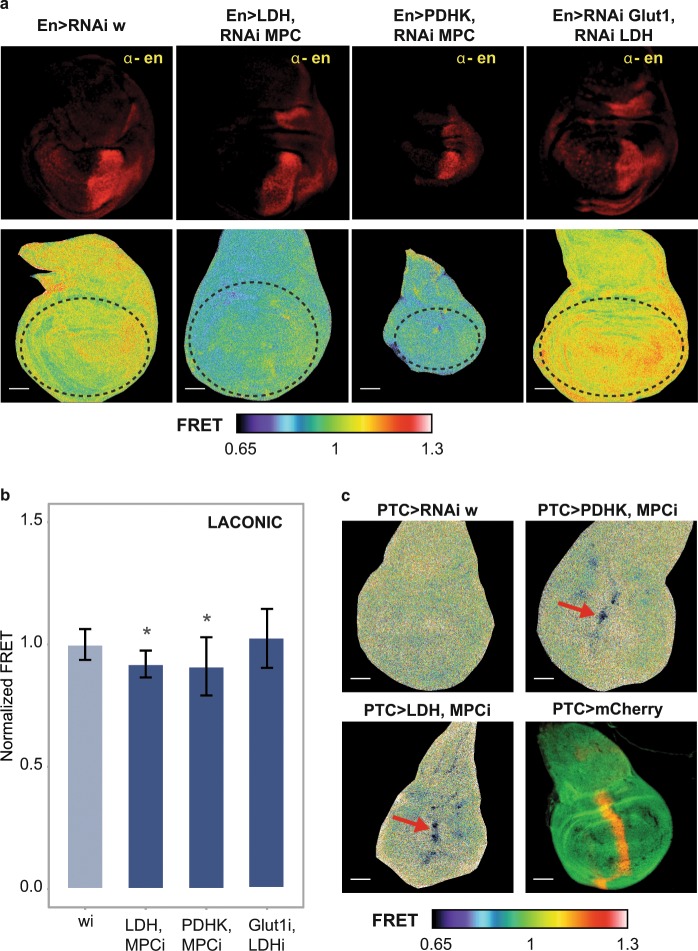
Tissue bioenergetic metabolism can be genetically rewired. (**a**,**b**) Laconic FRET maps and quantification of the FRET signal in larval wing discs, in which the expression of the indicated genes has been manipulated at the posterior compartment of the disc with en-Gal4. The upper panel shows the position of the posterior compartment, revealed by anti-En immunostaining. Dotted lines mark the region in which the FRET signal was determined. Data represent the media +/− SD; *p < 0.05 Dunnet’s Test; n ≥ 20 per group. (**c**) Laconic FRET maps of wing discs where the combined genetic manipulations indicated on each column were carried out at a restricted domain in the anterior/posterior frontier using the ptc-Gal4 driver. Red arrows mark groups of cells with increased lactate levels. The last panel shows the extension of the ptc domain as revealed by ptc > mCherry.

We analyzed the Laconic signal after manipulating the levels of the glucose transporter Glut1, the Mitochondrial Pyruvate Carrier (MPC), and the Monocarboxylate Transporters, (MCTs) Silnoon and Chaski. We also manipulated the expression of key enzymes of the energetic metabolism such as pyruvate kinase (PK), pyruvate dehydrogenase kinase (PDHK) or lactate dehydrogenase (LDH) (Fig. 1a). All the above genes are essential for regulation of the glycolysis/OXPHOS balance in diverse physiological or pathological contexts (Table 1). Neither silencing nor over-expression of any of these individual genes elicited alterations of the Laconic FRET signal in wing discs (Supplementary Fig. 4). In an attempt to overcome metabolic robustness, and force alterations of intracellular lactate levels, we induced combinations of two simultaneous genetic manipulations expected to act synergistically. As depicted in Fig. 5, silencing of MPC combined with overexpression of PDHK or LDH led to increased intracellular lactate levels in the cells where the expression of those genes was altered (Fig. 5a,b).

**Table 1:** Key metabolic enzymes or transporters whose transcriptional deregulation has been reported to alter the energetic metabolism.

Protein	Role in the energetic metabolism	Alterations reported in cancer contexts	References
GLUT1	Glucose transport through plasma membrane	Activation of isoforms 1 and 3 in mammalian tumor models. Its silencing, concomitant with LDH, reduces the tumor phenotype observed upon Notch activation in *Drosophila* imaginal discs	^34,38^
Pyruvate Kinase (PK)	Conversion of Phosphoenolpyruvate into pyruvate	Increase of a PKM2 isoform in several mammalian tumor models	^39,40^
Mitochondrial Pyruvate Carrier (MPC)	Mitochondrial transporters of pyruvate	Inhibition of isoforms 1 and 2 in mammalian tumor models. Its silencing in *Drosophila* leads to larval lethality when development occurs in media lacking a carbon source other than sucrose	^41,42^
Monocarboxylate Transporter (MCT)/Silnoon and Chaski	Transporters of lactate and pyruvate in the plasma membrane	Heightened expression of isoform 4 in mammalian tumor models	^43^
Pyruvate Dehydrogenase Kinase (PDHK)	Inhibition of the Pyruvate Dehydrogenase complex	Increase in Isoform 1 in several mammalian tumor models	^44,45^
Lactate Dehydrogenase (LDH)/Impl3	Reduction of pyruvate and lactate synthesis	Increase in LDHA isoform in several mammalian tumor models Transformation from hyperplasia to neoplasia depends on LDH activity	^37,46^

The table summarizes the physiological role of each of the genes in metabolism as well as the nature of the alteration in tumorigenesis.

It is noteworthy that, albeit genetic manipulations were performed in just the posterior domain of the wing imaginal discs (en-Gal4), variation of the Laconic signal was observed in the whole organ (Fig. 5a). This can be explained by rapid diffusion of lactate, as lactate, as well as pyruvate, diffuses very rapidly into and out from the cells through monocarboxylate transporters of the plasma membrane^1,16,28^. Thus it is not surprising that territories that are in proximity to highly lactogenic areas might also display increased levels of intracellular lactate. Next, we reasoned that, if lactate synthesis can be genetically increased in a more restricted territory, lactate levels might not be altered so globally, and a spatial heterogeneity of lactate levels might be detected in the wing disc. To this end, we utilized a Ptc-Gal4 driver to induce genetic manipulations, and indeed, increased lactate levels became evident specifically within a group of cells in this domain (Fig. 5c).

The energetic metabolism is a paradigmatic case of biological robustness^29,30^, and thus it is not surprising that the metabolic outcome is not altered after manipulation of single metabolic genes. For example, when lactate synthesis rates are below a certain threshold, MCTs may act as a drain for lactate, avoiding intracellular accumulation. If this is the case, at lactate levels that are above that threshold, flux through MCTs could become saturated, provoking intracellular lactate accumulation. Thus, the increased lactate levels that we observe by combining manipulations of the MPC and either LDH or PDHK might reflect this dynamics.

### The Warburg effect in *Drosophila*

**Figure 6:**
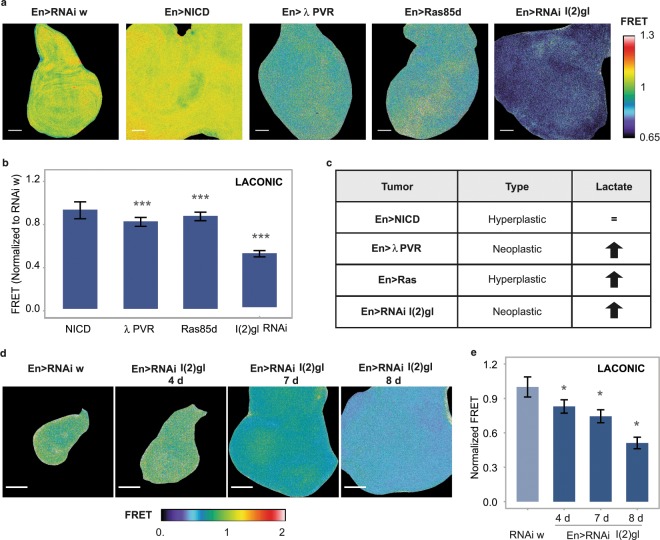
Heterogeneity of the metabolic state of tumors with various genetic backgrounds. (**a**,**b**) Laconic FRET maps and quantification of tumors induced through the indicated genetic manipulation in wing discs, using an en-Gal4 driver. Scale bar: 50 μm. Data represent the media +/− SD. The FRET signal of each tumor sample was quantified in the total area of the disc, and then normalized to w RNAi control discs. The media of each condition was statistically compared with its own control data set; p = 3.7394E-05 for PVR, p = 3.482E-05 for Ras and p = 6.6187E-15 for l(2)gl RNAi, Student’s T-test; n ≥ 20 per group. (**c**) Summary of the lactate levels in each of the tumors. (**d**) Laconic FRET maps of tumors induced by l(2)gl silencing with an en-Gal4 driver. As time passes after the onset of l(2)gl RNAi expression, lactic acid gradually accumulates. **(e)** Quantification of the results shown in panel (d). Data represent the media +/− SD; * p < 0.05 Dunnet’s Test; n ≥ 20 per group.

Even under conditions of oxygen availability, human tumor cells undergo a metabolic switch towards glycolysis, known as the Warburg effect^31^, and recent reports indicate that these metabolic alterations are recapitulated in *Drosophila* experimental tumors^32,33^. We tested whether Laconic can detect a Warburg-like metabolic switch in *Drosophila* experimental tumors generated by a variety of genetic strategies. Interestingly, and in line with earlier indirect observations^33^, only some specific genetic manipulations elicited the Warburg effect (Fig. 6a–c).

Constitutive activation of the Notch pathway in wing imaginal discs produces deregulated growth and hyperplastic tumors^34^, which depend on the induction of glycolysis-related genes such as LDH, Hexokinase A, and Glut1. Using Laconic, we analyzed intracellular lactate levels in these developing tumors, and found no significant differences in comparison to normal tissues (Fig. 6a,b). In contrast, overexpression of an activated variant of the PDGF/VEGF receptor homolog PVR^33^, or of an activated version of Ras, provoked a reduction of the Laconic FRET signal (Fig. 6a,b), indicating that lactate accumulated intracellularly. Tumors have a deeply altered physiology which could affect the function of the sensors. To confirm that the decrease of Laconic signal indeed reflects enhanced lactate levels in the tumor context, we treated the tumors *ex-vivo* with high concentrations of either glucose or 2-deoxyglucose (2-DG), a glucose analogue that cannot be catabolized via glycolysis. As expected, the Laconic FRET signal was higher in the discs exposed to 2-DG than in the controls treated with glucose (Supplementary Fig. 5), supporting the notion that lactate accumulates in these tumors.

The metabolic rewiring following Ras- or PVR-induced tumorigenesis is consistent with a previous report^33^, in which tumors induced through the same strategies displayed overexpression of LDH. We also employed Laconic to analyze tumor models in which metabolism had not been explored before. *Lethal (2) giant larvae* (l2gl) is a membrane-associated protein that regulates both cell proliferation and the epithelial polarity, and whose mutants have been shown to produce neoplastic tumors^35^. Silencing of l2gl led to the formation of lactogenic tumors (Fig. 6a,b). Interestingly, we found a tight temporal correlation between tumor development and lactate levels after the onset of l2gl silencing (Fig. 6d,e), suggesting that the metabolic status of the tumor is dynamic, and becomes increasingly lactogenic after the initial cell transformation.

Tumors in *Drosophila* are described as either *hyperplastic* or *neoplastic*. Whilst the former category is defined by deregulated proliferation with no alterations of cell shape or tissue polarity^36^, neoplastic tumors encompass rounded cells that lost polarization and associate with an altered architecture of the tissue^36^. It has recently been reported in tumors induced by overexpression of the EGF receptor^37^ that a lactogenic switch is associated to transformation of hyperplastic to neoplastic tumors. In line with this finding, our observations that the neoplastic tumors induced by l(2)gl or activated PVR undergo a lactogenic switch suggest that this metabolic requirement might be, in fact, a general feature of neoplastic growth. However, the fact that Ras-induced tumors are also lactogenic indicates that the metabolic switch is not restricted to neoplasia. Taken together, our results indicate that tumors of different genetic origin display different metabolic properties (Fig. 6a–c); a facet of tumor biology that is just starting to be explored.

### Final remarks

We have shown here that metabolic FRET sensors can be used to characterize the metabolic response of a single organ to stress conditions. We found not only that wing imaginal discs respond in a different manner whether the stress is induced by nutrient or oxygen deprivation, but also that different organs react differently to hypoxia. We have also shown that Laconic is capable of reporting an altered lactate concentration produced by genetic manipulation of key metabolic enzymes in a restricted domain of wing discs. These results pave the way to a systematic exploration of the effect of single genes on cell metabolic status *in vivo*. Reverse genetics analyses utilizing these metabolic sensors could shed light on the role that each enzyme and transporter plays in metabolic responses to different physiologic or pathologic conditions.

## Methods

### Fly lines and stocks

The following fly stocks were obtained from the Bloomington *Drosophila* Stock Center (https://bdsc.indiana.edu/): UAS-NICD (52008), UAS-λPVR (58428), UAS-Ras85d (4847) tub‐Gal4 (5138), en-Gal4 (1973), ptc-Gal4 (2017), Glut1 RNAi (40904) l(2)gl RNAi (38989) and white RNAi (33613). The following stocks were from the Vienna *Drosophila* Research Center (https://stockcenter.vdrc.at/): LDH RNAi (110190), PDHK RNAi (37966), MPC RNAi (103829), PK RNAi (49533) Chk (MCT) RNAi (37141) and Silnoon (MCT) RNAi (106773). The UAS-LDH line was obtained from the Zurich ORFeome Project (https://flyorf.ch/).

The following lines were generated in this work: UAS-Laconic, UAS-Pyronic, UAS-OGsor, tub-Laconic, UAS-PDHK.

### Cloning and transgenic lines generation

Transgenic lines bearing UAS-Laconic, UAS-Pyronic, UAS-OGsor and tub-Laconic were generated by phiC31-mediated site-directed integration on the 58A landing site. UAS-PDHK, on the other hand, was integrated into the 86F landing site.

The ORF of Laconic and Pyronic were subcloned into the pUASt.attB vector using XhoI and XbaI. OGsor was subcloned in the same vector using BglII and NotI. Laconic was also subcloned into pss193, downstream of the tubulin promoter, using BamHI and XbaI. Finally, the ORF of PDHK was obtained from the *Drosophila* Genomics Resource Center (https://dgrc.bio.indiana.edu; #BS06809), and then subcloned into pUASt.attb using NotI and XbaI.

### Microscopy setup and image acquisition

Images of the three relevant channels were obtained simultaneously using the QUASAR detection unit of the Zeiss 710 or 880 microscopes. The emission windows that define these channels are: donor (CFP) channel, 490 +/− 5 nm; acceptor (YFP) channel, 530 +/− 5 nm; and autofluorescence (A) channel, 600 +/− 5 nm. Emission spectra, whenever required, were obtained using a Zeiss LSM510 Meta Confocal Microscope with monochromator. Sensors were excited at 405 nm or 458 nm. FRET maps were built as explained in the supplementary material section. To obtain quantitative values, the FRET signal was measured in specific regions of the organs. These regions are marked with dotted lines in representative images shown in the Figures.

### Starvation and hypoxia treatment

For larval starvation, 3^rd^ instar larvae were collected from their regular medium, and placed in 2% agar plates supplemented with 3% sucrose, for 6 h as previously reported^27^. For hypoxia treatments the proportions of oxygen and nitrogen were regulated in a Forma Scientific 3131 incubator. Third instar larvae were subjected to hypoxia for 16 h before dissection and observation under the confocal microscope. Larvae were dissected in PBS and then fixed in 4% formaldehyde (Sigma, St. Louis, MO, USA) for 120 min at room temperature. After washing three times for 10 minutes in PT (PBS, 0.3% Triton-X 100), the required organs were separated and mounted in Mowiol (Calbiochem, Merck & Co, New Jersey, USA).

### Statistical analysis

Data are expressed as mean ± standard deviation (SD). When comparing between two conditions, the Student’s T-test was employed. Normality was tested using the Shapiro–Wilks test. If data did not followed a normal distribution, the Mann-Whitney test was used instead. For multiple comparisons, one-way analysis of variance (ANOVA) followed by Dunnet’s test was performed; in this case, data were tested for normality with the Shapiro–Wilks test and variance homogeneity with the Levene test. Box-Cox transformations were employed whenever normality or homocedasticity requirements were not satisfied. A p < 0.05 was considered statistically significant. Statistical analyses were executed using GraphPad Prism, version 5.03 (GraphPad Software).

## Supplementary information

Supplementary Information

Supplementary Video

## Data Availability

The datasets generated during the current study are available from the corresponding author on reasonable request.
