# Supplementary material for: A genetic toolkit for the analysis of metabolic changes in Drosophila provides new insights into metabolic responses to stress and malignant transformation
Source: Sci Rep. 2019 Dec 27;9:19945. doi: 10.1038/s41598-019-56446-3 (PMC6934733; doi:10.1038/s41598-019-56446-3)
Supplement: Supplementary file 1 — Supplementary Information [file 41598_2019_56446_MOESM1_ESM.pdf]

Supplementary Information

A genetic toolkit for the analysis of metabolic changes in *Drosophila* provides new insights into metabolic responses to stress and malignant transformation

Gándara L[1,3], Durrieu L[1, 2], Behrensen C[1], Wappner P[1, 2,3*].

[1]Instituto Leloir, Ciudad de Buenos Aires, Argentina
[2]Departamento de Fisiología, Biología Molecular, y Celular, Facultad de Ciencias Exactas y Naturales, Universidad de Buenos Aires, Ciudad de Buenos Aires, Argentina
[3]Consejo Nacional de Investigaciones Científicas y Técnicas (CONICET)

*Corresponding author: pwappner@leloir.org.ar

Supplementary Methods - Utilization of a linear unmixing algorithm to correct background and autofluorescence in FRET images

Autofluorescence is usually assumed to be a constant –and minor- component of the aquired fluorescence signal. However, this is not the case in *Drosophila* tissues that -due to their composition and complexity- show high and inhomogeneous autofluorescence (Sup Fig 1f). Autofluorescence is particularly problematic when assessing signal ratios, such as those characteristic of FRET experiments, especially when the intensity is low. In the current work, we estimated the autofluorescence in the donor and acceptor emission channels utilizing a spectral unmixing algorithm, followed by pixel-by-pixel substraction of the autofluorescence value obtained. The assumption in this method is that the fluorescence measured in a given channel (for example, YFP, I_m^Y) is the result of the sum of the fluorophore signal (I_f^Y) plus autofluorescence (in this example, detected in the YFP channel) (I_{af}^Y) (eq 1).

$$I_m^Y(x,y) = I_f^Y(x,y) + I_{af}^Y(x,y) \qquad (1)$$

Since direct determination of I_{af}^Y is not possible due to its overlap with I_f^Y, I_{af}^Y is estimated from the fluorescence intensity in a different emission wavelength, where no fluorophore signal can be detected. We call this third channel, defined by donor excitation wavelength and only autofluorescence emission, "A". I_{af}^A can be then directly measured by taking a dedicated image in this particular channel (no additional illumination is necessary).

Estimation of the fluorescence intensity in the YFP channel (I_{af}^Y) from the autofluorescence signal in the AF channel (I_{af}^A) is possible due to the property of autofluorescence of having a wide emission spectrum that is reproducible for a given tissue. This happens because the source of autofluoresence is a complex mixture of different molecules (*40*), in contrast to a single fluorophore such as YFP. Thus the autofluorescence emission spectrum can be characterized in un-labeled samples, establishing a relation between the measured intensity in the YFP (I_{af}^Y) and the AF (I_{af}^A) channels that will be maintained in labeled samples. This method assumes that the relation between the fluorescence intensities detected in both channels is linear. Estimation of I_{af}^Y is then performed by weighting the I_{af}^A by a constant K (eq 2).

$$I_{af}^Y(\mathrm{x,y}) = \mathrm{K} * I_{af}^A(x,y) \qquad (2)$$

Noteworthy, while the fluorescence intensity from the fluorophore (I_{af}^{Y}), and autofluorescence (I_{af}^{A}) are functions of space, the constant K depends only on tissue properties and image settings. Then, eq.1 can be combined with eq 2, to get the correction equation (eq 3).

$$I_f^Y(x,y) = I_m^Y(x,y) - K * I_{af}^A(x,y) \qquad (3)$$

To apply this algorithm, then it is necessary to *i*) estimate K, and *ii*) acquire a specific image, I_{af}^{A} in each experiment. For characterization of the autofluorescence emission spectrum and estimation of K, images from each of the three channels were obtained from tissues that do not express any sensor –where all the emission arises from autofluorescence-.

When working with sensor-expressing tissues, the autofluorescence was corrected according to eq. 3, and the ratiometric FRET signal (F) was calculated as in eq. 4

$$F(x,y) = \frac{I_f^Y(x,y)}{I_f^C(x,y)} \qquad (4)$$

By using eq 4, it is possible to calculate a FRET signal for each pixel, thereby generating the FRET map of the sample.

Sup Fig 1

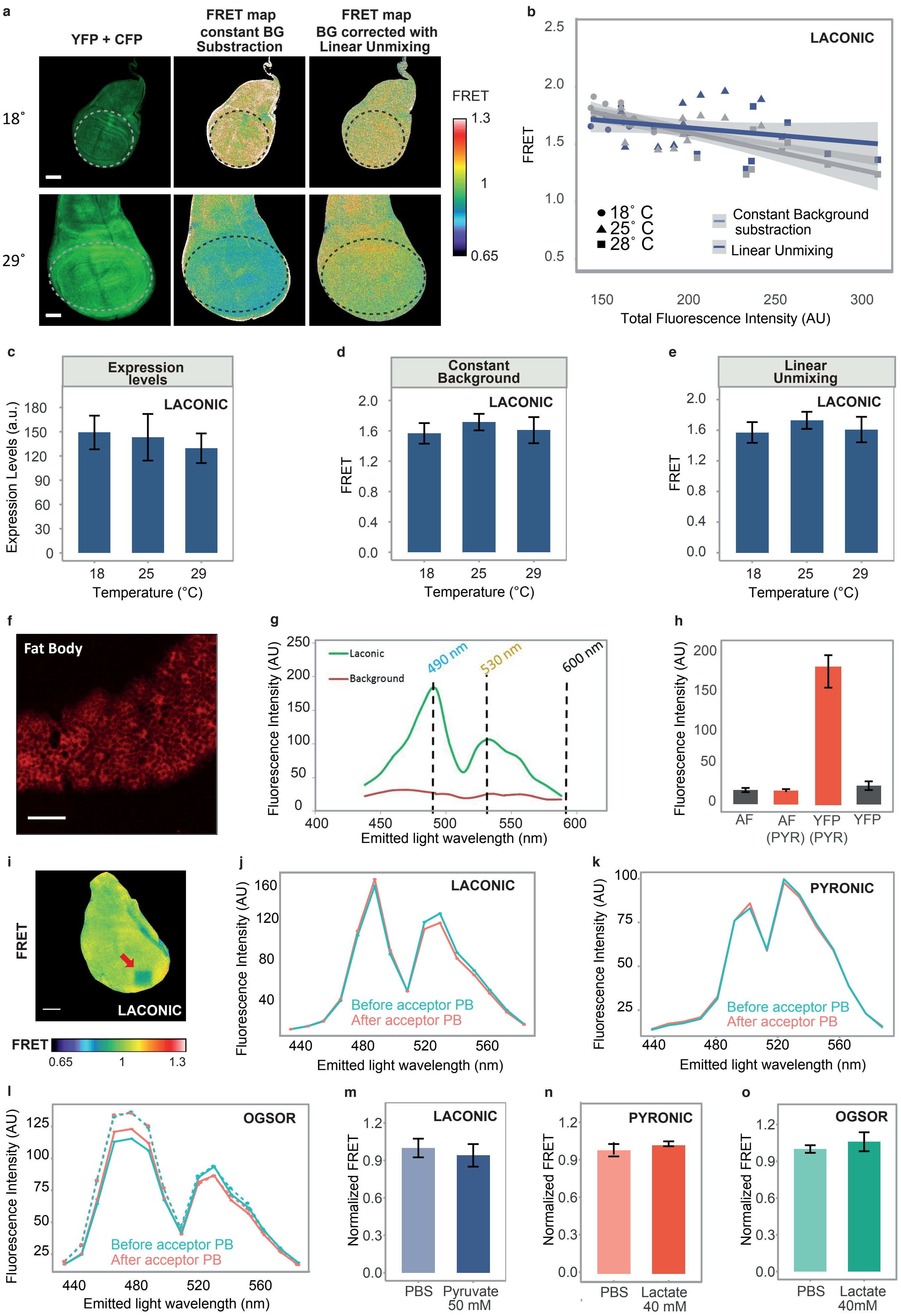

a
YFP + CFP
FRET map constant BG Substraction
FRET map BG corrected with Linear Unmixing
18˚
29˚
FRET
1.3
1
0.65
b
LACONIC
FRET
2.5
2.0
1.5
1.0
0.5
18˚ C
25˚ C
28˚ C
Constant Background substraction
Linear Unmixing
150
200
250
300
Total Fluorescence Intensity (AU)
c
Expression levels
LACONIC
Expression Levels (a.u.)
180
150
120
90
60
30
0
18
25
29
Temperature (°C)
d
Constant Background
LACONIC
FRET
2.0
1.6
1.2
0.8
0.4
0.0
18
25
29
Temperature (°C)
e
Linear Unmixing
LACONIC
FRET
2.0
1.6
1.2
0.8
0.4
0.0
18
25
29
Temperature (°C)
f
Fat Body
g
Laconic
Background
490 nm
530 nm
600 nm
Fluorescence Intensity (AU)
250
200
150
100
50
0
400
450
500
550
600
Emitted light wavelength (nm)
h
Fluorescence Intensity (AU)
200
150
100
50
0
AF
AF (PYR)
YFP (PYR)
YFP
i
FRET
LACONIC
FRET
0.65
1
1.3
j
LACONIC
Fluorescence Intensity (AU)
160
120
80
40
Before acceptor PB
After acceptor PB
440
480
520
560
Emitted light wavelength (nm)
k
PYRONIC
Fluorescence Intensity (AU)
100
75
50
25
Before acceptor PB
After acceptor PB
440
480
520
560
Emitted light wavelength (nm)
l
OGSOR
Fluorescence Intensity (AU)
125
100
75
50
25
Before acceptor PB
After acceptor PB
440
480
520
560
Emitted light wavelength (nm)
m
LACONIC
Normalized FRET
1.2
0.9
0.6
0.3
0.0
PBS
Pyruvate 50 mM
n
PYRONIC
Normalized FRET
1.2
0.9
0.6
0.3
0.0
PBS
Lactate 40 mM
o
OGSOR
Normalized FRET
1.2
0.9
0.6
0.3
0.0
PBS
Lactate 40mM

Supplementary Figure 1. Validation of the FRET signals. (**a**) FRET maps of the Laconic signal of wing imaginal discs in which different expression levels of the sensor were attained by using a tub-Gal4 driver at 25º C or 29º C. Note that the FRET signal obtained without linear unmixing is dependent on the expression levels of the sensor, while this dependency is largely suppressed after applying the linear unmixing algorithm. A high FRET signal in the color code shown on the right is associated to lower lactate levels. Scale bar: 50 μm. Dotted lines mark the region in which the FRET signal was measured. (**b**) When a constant autofluorescence background value was subtracted throughout the image, the apparent FRET map (artefactual) apparently depends on expression levels of the sensor (grey curve; p= 1.07e-05). However, when the linear unmixing algorithm was utilized, the FRET map is no longer dependent on the expression levels of the sensor (blue curve; p= 0.114). (**c-e**) Temperature does not alter lactate levels *per se*. When Laconic is expressed with a constitutive, temperature-independent driver, its expression levels are, as expected, not modified (**c**), and the FRET signal remains unaltered regardless of background substraction method utilized (**d**, **e**). (**f**) Autofluorescence at 600 nm of a wild type fat body upon excitation at 405 nm. Scale Bar: 50 μm. (**g**) Emission spectra of wild type (red) or Laconic-expressing (green) wing discs upon excitation at 405 nm. The three channels relevant to the linear unmixing algorithm are highlighted with vertical dotted lines: CFP-donor channel (490 nm), YFP-acceptor channel (530 nm) and autofluorescence (AF) channel (600 nm). (**h**) Fluorescence intensity at 600 nm of wild type wing discs (AF), or of Pyronic-expressing discs (AF - Pyr), at 530 nm of Pyronic-expressing discs (YFP – Pyr) or of YFP-expressing discs (YFP), upon illumination at 458 nm. (**i**) Laconic FRET map of a wing disc in which YFP has been photobleached by irradiating with high fluence at 488 nm in a small region of the posterior compartment (arrow). Scale bar: 50 μm. (**j**) Emission spectra of Laconic-expressing discs upon excitation at 458 nm before or after YFP photobleaching. (**k**) Emission spectra of Pyronic-expressing discs upon excitation at 458 nm before or after YFP photobleaching. (**l**) Emission spectra of OGsor-expressing discs upon excitation at 458 nm before or after YFP photobleaching, either in fixed (dotted lines) or unfixed samples (solid lines). (**m**) Laconic signal of wing imaginal discs incubated for 15 min either in PBS or in a 50mM pyruvate solution. Data represent the media +/- SD. (**n-o**) Pyronic (**n**) or OGsor (**o**) signal of wing imaginal discs incubated for 15 min either in PBS or in a 40mM lactate solution. Data represent the media +/- SD.

Sup Fig 2

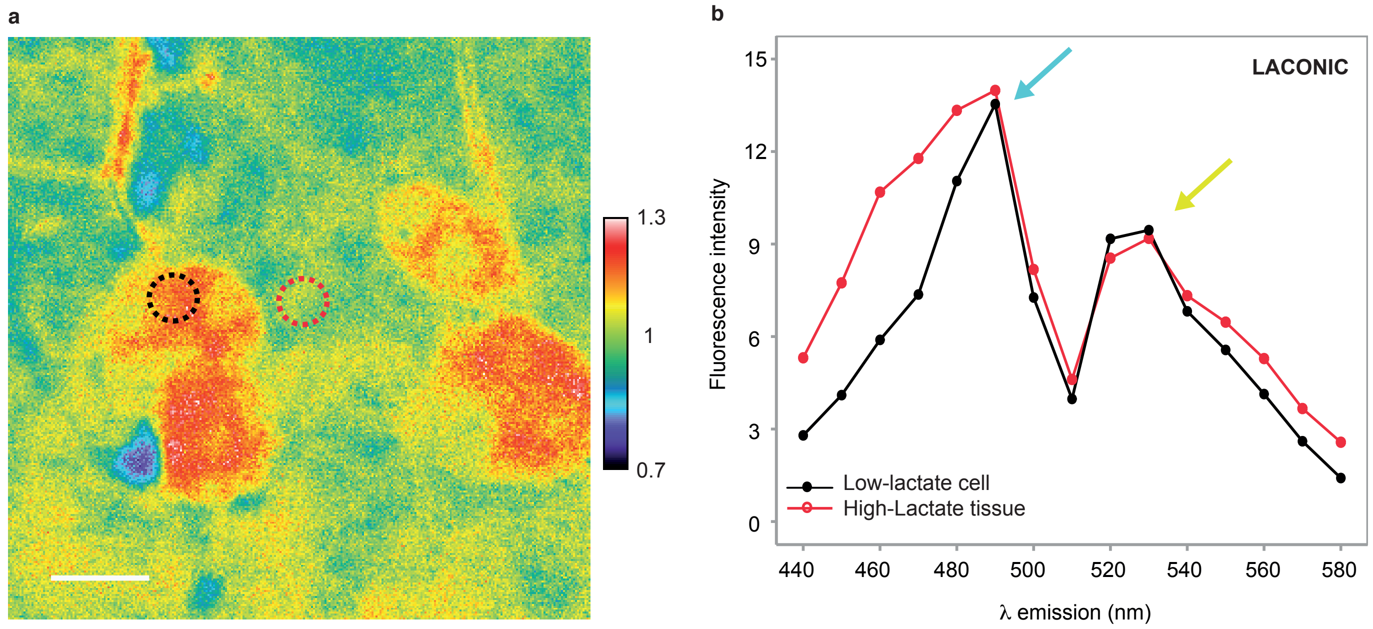

a
1.3
1
0.7
b
LACONIC
Fluorescence intensity
15
12
9
6
3
0
440
460
480
500
520
540
560
580
λ emission (nm)
Low-lactate cell
High-Lactate tissue

Supplementary Figure 2. (a) Cells of the larval nervous system displaying different lactate levels (magnification of the area marked in Figure. 2a, Brain, dotted square). The quantified regions within a cell with low lactate levels (black circle) and a neighboring cell with higher lactate levels (red circle) are shown. Scale bar= 10 μm. **(b)** The emission spectrum of the cell with low lactate levels (black) is compared with the spectrum of a cell with higher lactate levels (red). The low-lactate cell shows decreased CFP (cyan arrow) and enhanced YFP emission (yellow arrow), indicating that the increase of the FRET signal in the hypolactic cell is reliable.

Sup Fig 3

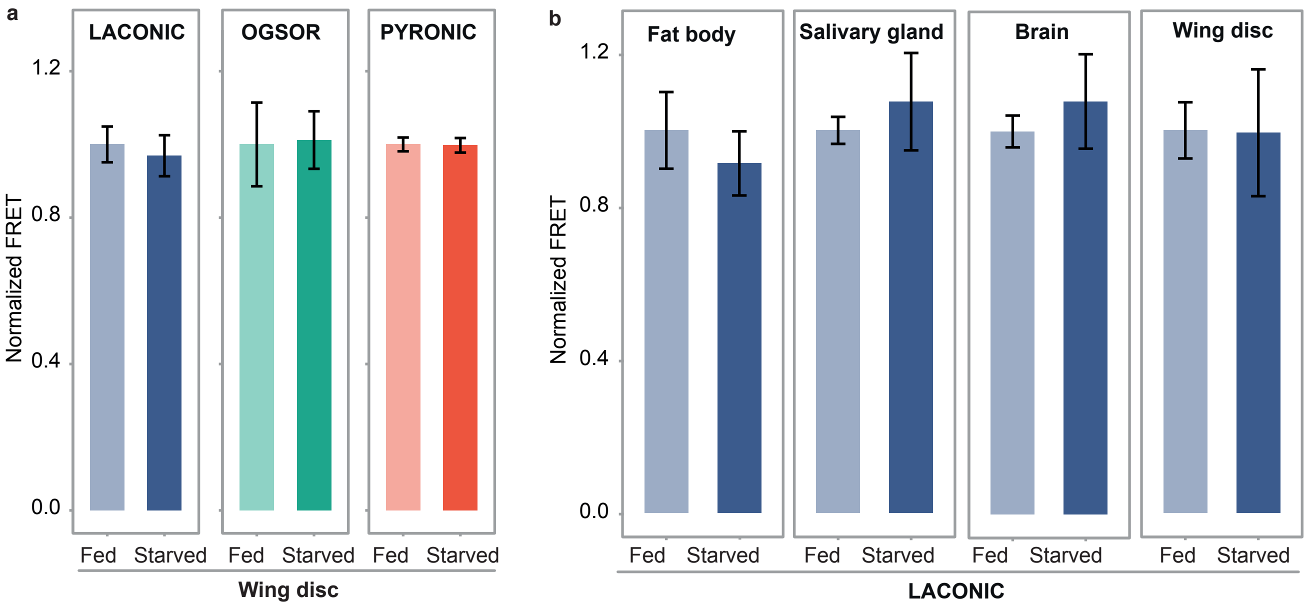
a
LACONIC
OGSOR
PYRONIC
Normalized FRET
1.2
0.8
0.4
0.0
Fed
Starved
Fed
Starved
Fed
Starved
Wing disc
b
Fat body
Salivary gland
Brain
Wing disc
Normalized FRET
1.2
0.8
0.4
0.0
Fed
Starved
Fed
Starved
Fed
Starved
Fed
Starved
LACONIC

Supplementary Figure 3. Metabolite levels remain constant in wing discs of larvae are subjected to nutrient deprivation. (**a**) Laconic, Pyronic and OGsor FRET signals from wing imaginal discs of 3rd instar larvae fed *ad libitum* or subjected to 6 h starvation. No differences in the levels of Lactate, Pyruvate or 2-OG could be detected. (**b**) Laconic FRET signal from 3rd instar larval wing disc, brain, salivary gland and fat body are shown under fed or starvation (18 h) conditions. No differences in lactate levels could be detected. Data represent the media +/- SD. n≥20 per group.

Sup Fig 4

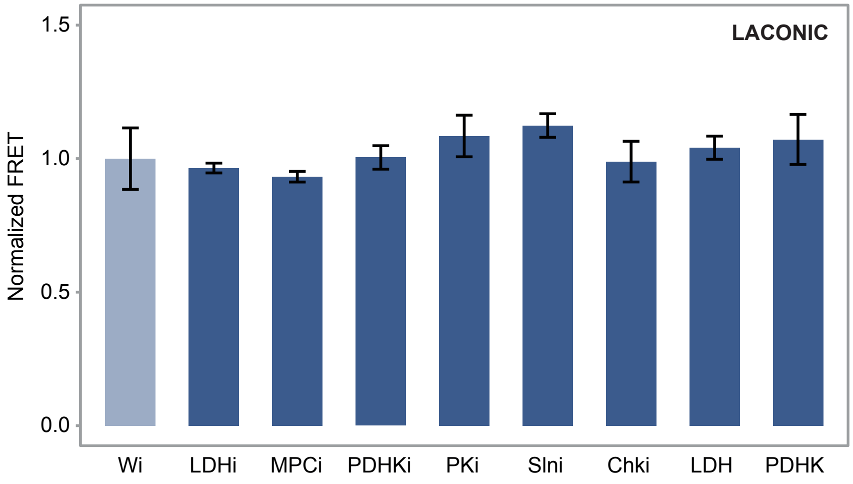

LACONIC
Normalized FRET
1.5
1.0
0.5
0.0
Wi
LDHi
MPCi
PDHKi
PKi
SIni
Chki
LDH
PDHK

Supplementary Figure 4. Single genetic manipulations do not alter lactate levels. Laconic FRET signal of wing discs where the indicated proteins were either overexpressed or silenced by expression of specific RNAis with an En-Gal4 driver. None of the single genetic manipulations was sufficient to alter lactate levels. Data represent the media +/- SD. $n \geq 20$ per group.

Sup Fig 5

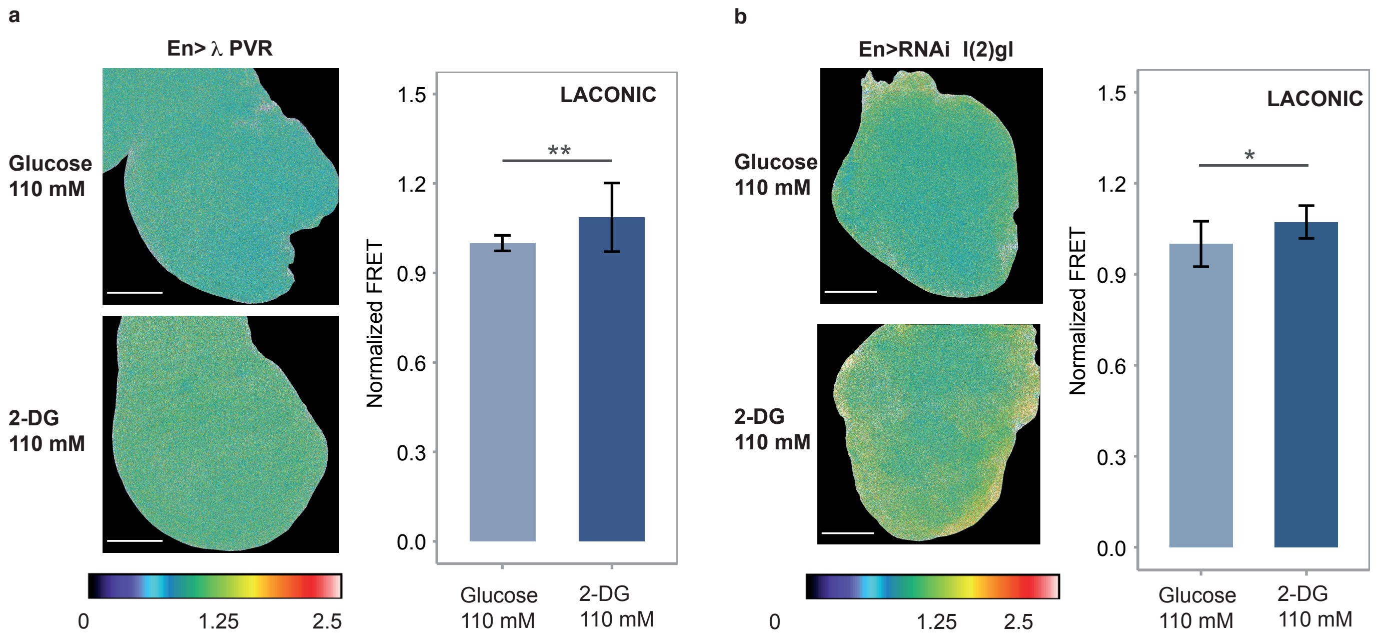
a
En> λ PVR
Glucose 110 mM
2-DG 110 mM
0
1.25
2.5
LACONIC
Normalized FRET
1.5
1.2
0.9
0.6
0.3
0.0
**
Glucose 110 mM
2-DG 110 mM
b
En>RNAi l(2)gl
Glucose 110 mM
2-DG 110 mM
0
1.25
2.5
LACONIC
Normalized FRET
1.5
1.2
0.9
0.6
0.3
0.0
*
Glucose 110 mM
2-DG 110 mM

Supplementary Figure 5. The laconic sensor reports a *bona fide* increase of lactate levels in experimental tumors. (**a**) Laconic FRET signal in En> λ PVR tumors on which glycolysis has been impaired or not by incubation with 2-DG or with glucose (control). Tumorous discs treated with 2-DG display a higher FRET signal than glucose-incubated discs (quantification on the right). (**b**) Similar to (**a**), but in a tumor induced by En-Gal mediated l(2)gl RNAi expression (quantification on the right)**.** For quantifications the FRET signal from whole discs was recorded. Scale bar= 100 μm. Data represent the media +/- SD. p= 0.0041 for λ PVR and p=0.0215 for l(2)gl RNAi, Student's T-test; n≥10 per group.
